# Uterine Fibroma

**Published:** 1893-03

**Authors:** J. B. Murfree

**Affiliations:** Murfreesboro, Tenn.


### UTERINE FIBROMA.

By J. B. MURFREE, A.M., M.D.,
MURFREESBORO, TENN.

Every part of the human body is supplied with nerves, blood-vessels, capillaries and lymphatics, and there is a continuous growth. Under the general law of nutrition there is regularly being added new material, and there is also a constant waste of tissue elements. In the adult, waste and repair is nearly equal. Occasionally from some disturbance of this equalization, the accretion of nutrient material is in excess, producing a surplus of tissue, which again is subject to accretion.

A fibroid tumor is fed by the same blood that nourishes the normal tissues of the uterus. A uterine fibroma is a morbid growth developed within the walls of the uterus, and is composed of muscular fibro-cells, fibro-plastic material and cellular tissue, and is due to a perversion of nutrition, is non-malignant and homologous in its structure. They have existed in all ages, but were not definitely recognized until within the present century. Their histological elements are the same as those of the uterus, but differ in the construction and arrangement of the textures, the fibroma being denser, less vascular and less colored.

The usual seat of a fibroma is the body and fundus of the womb, and while they are innocent and have no tendency to malignancy, yet they cause a great deal of distress by their mechanical pressure. Pain, hemorrhages, rectal and cystic irritations, indigestion, dropsy and exhaustion are some of their results, and sometimes produce death. While a uterine fibroma is not malignant, yet it is progressive. A fibrous tumor originates within the muscular walls of the uterus, but the direction of its growth may vary. When it is within the walls of the uterus it is *interstitial*, when it grows towards the external surface it is *sub-peritoneal*, and when toward the internal surface, *submucous*. The interstitial is stationary and gives the least trouble. Sometimes from different causes these tumors gradually disappear entirely, or sloughing, may be expelled through the vagina. Sometimes they soften in the interior and become fibro-cystic. The period of the appearance of fibroid tumors is middle life. They are unknown before puberty, and never originate after the menopause. They are retarded in their development by child-bearing,

The unmarried woman is most liable to have uterine fibroma, the sterile less so, and the fruitful the least. In some way this abnormal growth is connected with the exercise of the functions of the uterus.

The prognosis is usually favorable, although they cause much suffering, and sometimes death, yet often they remain stationary or entirely disappear, and if the woman lives, they rapidly decrease after the monopause. They threaten life by (1) hemorrhage, (2) inflammation, (3) septicæmia, (4) pressure. The prognosis is also rendered more favorable by the advances made in the treatment of these tumors, both medical and surgical.

The pathology of uterine fibroma consists in an increase in the size of the uterus, in the formation of mucous polypi and in inflammation of the mucous membrane. The uterus is disturbed in its axis and position and encroaches upon adjacent organs.

The symptoms are a disturbance of the menstruation, either too frequent or too profuse, leucorrhœas, constipation, dysuria, enlargement, debility. The physical signs are displacement and hardening of the os while there is an irregular enlargement of the uterus, with the lengthening of its cavity.

The treatment of uterine fibroma is divided into four methods.

(1) Symptomatic. (2) General (by medicine). (3) Electrolysis. (4) Surgical.

By the first method we simpiy treat the symptoms as they arise and ward off threatened dangers. Hemorrhage is the most troublesome symptom and is best treated by quietude, opiates, the hot douche and the tampon. The general treatment of uterine fibroma is by the administration of medicine to cause the absorption or destruction of the tumor. Medicines are powerless to cause the absorption of a fibrous tumor and do good, except to build up the general system. Ergot is given to cause the death and expulsion of the tumor. I have no confidence in ergot. The treatment by electrolysis has met with some success and is worthy of trial. It is especially adapted to the interstitial variety. Surgical treatment is most usually resorted to for the permanent relief of uterine fibroma. It consists in the removal of the tumor by traction, torsion, excision, enucleation, ecrasement and hysterectomy. When the tumor projects into the uterine cavity it is removed by excision. When it is interstitial it should be treated by electricity. When sub-peritoneal it had better be left alone unless the woman's life is a burden and death is threatening, when it should be removed through a laparotomy or by hysterectomy. Hysterectomy should never be resorted to as an ideal operation, but only as a forlorn hope. But conditions do arise when it is eminently proper and should unhesitatingly be performed.

